# Dosimetric consequences of translational and rotational errors in frame-less image-guided radiosurgery

**DOI:** 10.1186/1748-717X-7-63

**Published:** 2012-04-24

**Authors:** Matthias Guckenberger, Johannes Roesch, Kurt Baier, Reinhart A Sweeney, Michael Flentje

**Affiliations:** 1 Department of Radiation Oncology, University of Würzburg, Würzburg, Germany

**Keywords:** Radiosurgery, Frame-less, Frame-based, Stereotactic, Image-guidance

## Abstract

**Background:**

To investigate geometric and dosimetric accuracy of frame-less image-guided radiosurgery (IG-RS) for brain metastases.

**Methods and materials:**

Single fraction IG-RS was practiced in 72 patients with 98 brain metastases. Patient positioning and immobilization used either double- (n = 71) or single-layer (n = 27) thermoplastic masks. Pre-treatment set-up errors (n = 98) were evaluated with cone-beam CT (CBCT) based image-guidance (IG) and were corrected in six degrees of freedom without an action level. CBCT imaging after treatment measured intra-fractional errors (n = 64). Pre- and post-treatment errors were simulated in the treatment planning system and target coverage and dose conformity were evaluated. Three scenarios of 0 mm, 1 mm and 2 mm GTV-to-PTV (gross tumor volume, planning target volume) safety margins (SM) were simulated.

**Results:**

Errors prior to IG were 3.9 mm ± 1.7 mm (3D vector) and the maximum rotational error was 1.7° ± 0.8° on average. The post-treatment 3D error was 0.9 mm ± 0.6 mm. No differences between double- and single-layer masks were observed. Intra-fractional errors were significantly correlated with the total treatment time with 0.7mm±0.5mm and 1.2mm±0.7mm for treatment times ≤23 minutes and >23 minutes (p<0.01), respectively. Simulation of RS without image-guidance reduced target coverage and conformity to 75% ± 19% and 60% ± 25% of planned values. Each 3D set-up error of 1 mm decreased target coverage and dose conformity by 6% and 10% on average, respectively, with a large inter-patient variability. Pre-treatment correction of translations only but not rotations did not affect target coverage and conformity. Post-treatment errors reduced target coverage by >5% in 14% of the patients. A 1 mm safety margin fully compensated intra-fractional patient motion.

**Conclusions:**

IG-RS with online correction of translational errors achieves high geometric and dosimetric accuracy. Intra-fractional errors decrease target coverage and conformity unless compensated with appropriate safety margins.

## Background

Single fraction radiosurgery (RS) of intracranial malignant and benign lesions requires maximum accuracy of treatment planning and delivery to ensure that the irradiation doses are confined precisely to the target structures. For decades this accuracy of treatment delivery has been achieved by using invasive frame-based stereotactic systems: invasive fixation of the external stereotactic system to the patients’ skull and treatment on the same day without its detachment achieved precise localization of the patient and simultaneously effective patient immobilization.

Since several years, in-room image-guidance has become broadly available allowing frame-less image-guided radiosurgery (IG-RS) without the need for external stereotactic reference systems. This image-guided approach provides a fully noninvasive treatment option and has been systematically optimized in the recent years. The two key technologies of orthogonal planar x-rays [1,2] and cone-beam CT (CBCT) [3,4] solutions were shown to achieve sub-millimeter accuracy in phantom studies. No obvious differences in accuracy have been described despite both image-guidance approaches have specific pros and cons: whereas planar x-ray solutions allow for fast and repetitive intra-treatment imaging, even during non-coplanar beam delivery, volumetric image information is missing. Robotic couches have been developed which correct set-up errors with sub-millimeter residual errors and also allow correction of rotational errors [5-7]. Finally, various immobilization systems– e.g. thermoplastic masks or bite-block systems - have been described for patient immobilization and intra-fractional motion was usually reported to be within 1-2 mm [8]. Consequently, intra-fractional patient motion is considered as the weakest link in frame-less IG-RS.

Previous studies in the literature described the accuracy of frame-less IG-RS with geometric data only, which is a suboptimal endpoint from a clinical perspective. The consequences of set-up errors or intra-fractional patient motion on doses to the target volume and organs-at-risk will vary depending on the dose gradient, which itself depends on multiple factors like the radiotherapy delivery device (Gammaknife versus linear accelerator), multi-leaf collimator characteristics, beam set-up and size/shape of the target volume. Consequently, it is the aim of this study to evaluate the dosimetric consequences of set-up errors and intra-fractional patient motion during IG-RS. Additionally, the value of correcting rotational set-up errors was investigated because there exists no consensus in the literature whether this is necessary or not.

## Material and methods

### Patient and treatment characteristics

Between 2007 and 2011, 72 patients were treated for 98 brain metastases using frameless IG-RS at our department. Agreed consent was obtained by all patients.

This retrospective planning study used the original treatment plans for simulation of set-up errors and intra-fraction errors in IG-RS.

Patient immobilization was performed using thermoplastic masks in all patients (Unger Medizintechnik, Mülheim-Kärlich, Germany). In the first 71 cases, a double-layer thermoplastic mask was used to increase its rigidity. In the last 27 cases, a conventional single-layer mask was used. Treatment planning was based on co-registered CT with 2 mm slice thickness and volumetric MRI datasets using the Pinnacle treatment planning system (Philips Radiation Oncology Systems, Milpitas, USA). The gross tumor volume (GTV) was defined as the contrast enhanced region in the CT and MRI images. The planning target volume (PTV) was generated with safety margins of 1-2 mm at the discretion of the responsible physician. Treatment planning and dose prescription was independent from the applied safety margin and the size of the safety margin did not affect the results of this simulation study. Multiple coplanar and non-coplanar arcs or static beams were planned with dose prescription to the PTV surrounding 80% isodose line; grid size for dose calculation was 2 mm using collapsed cone convolution algorithm. Treatments were planned for an Elekta Synergy S linear accelerator equipped with the beam modulator with 4 mm leaf width (Elekta, Crawley, England).

For image-guided treatment delivery, patients were positioned using conventional drawings on the thermoplastic mask. A kilo-voltage CBCT was acquired with the patient in treatment position and automatic image-registration was performed between the planning CT and the verification CBCT in the XVI software (n = 98): the clipbox defining the region of interest for image registration excluded the neck and was confined to the skull in all patients. The correction reference point was located in the isocenter, which was the geometric center of the PTV. Translational and rotational errors were recorded in left-right (LR), anterior-posterior (AP) and superior-inferior (SI) direction. All translational and rotational errors were corrected without an action level by the robotic HexaPOD treatment couch. After treatment was finished, a CBCT was acquired for analysis of intra-fractional uncertainties (n = 64).

### Evaluation of IG-RS

Dosimetric accuracy of IG-RS was simulated within the Pinnacle treatment planning system using the patient specific treatment plans: three scenarios were evaluated: 1) simulation of translational and rotational set-up errors foranalysis of RS immediately after patient positioning without IG; 2) simulation of rotational set-up errors for analysis of IG-RS with correction of translational errors only; 3) simulation of translational and rotational post-treatment errors for analysis of IG-RS as clinically practiced in our department.

Translational and rotational errors were simulated within the Pinnacle treatment planning system by translating and rotating the planning CT data set including the planning contours relative to the treatment plan, which remained fixed in space.

Two dosimetric parameters were evaluated for the PTV. Coverage index (CovI) of the PTV was calculated based on the volume of the PTV (PTV) and the volume of the PTV, which received the prescription dose (PTV_PD_):

(1) $${\text{CovI}}_{\text{PTV}}={\text{PTV}}_{\text{PD}}/\text{PTV}$$

The Paddick coverage index (CI) [9] was calculated based on the PTV, PTV_PD_ and the total volume irradiation with the PD (Vol_PD_):

(2) $${\text{CI}}_{\text{PTV}}=\left({\text{PTV}}_{\text{PD}}/\text{ PTV}\right)\times \left({\text{PTV}}_{\text{PD}}/{\text{ Vol}}_{\text{PD}}\right)$$

Analysis of PTV coverage simulated treatment without any safety margins to the GTV, as practiced in traditional cranial radiosurgery. Additionally, we simulated the effects of safety margins by generation of two additional target volumes based on the PTV with negative isotropic margins of 1 mm (PTV-1 mm) and 2 mm (PTV-2 mm). This can be interpreted as analysis of GTV dose coverage while treatment planning was based on a PTV with GTV-to-PTV safety margins of 1 mm and 2 mm.

### Statistics

Statistica X (Statsoft, Tulsa, USA) was utilized. The Spearmen’s rank test was used for test of correlation and Student`s t test for comparison of dosimetric and geometric results between groups. Differences were considered significant for p < 0.05.

## Results

### Geometric accuracy of IG-RS

Set-up errors prior to IG are summarized in Table 1. The 3D set-up error was 3.9 mm ± 1.9 mm and almost 12 mm at maximum. Rotational errors were largest around the LR axis and the maximum rotational error around the three axes in each patient was 1.7° ± 0.8° on average. There was no significant difference in set-up errors – translations and rotations - between the single- and double-layer masks (p = 0.71).

**Table 1:** Patient positioning errors prior to cone-beam CT based image-guidance (IG) and immediately following image-guided radiosurgery (IG-RS)

	**Prior IG (n = 98)**			**Post IG-RS (n = 64)**		
	**Average**	**StDev**	**Max**	**Average**	**StDev**	**Max**
**LR [mm]**	0.1	2.1	10.3	0.1	0.6	1.8
**SI [mm]**	−0.8	1.7	5.4	−0.3	0.8	3.0
**AP [mm]**	−2.7	2.0	9.9	−0.2	0.4	1.4
**3D vector [mm]**	3.9	1.9	11.9	0.9	0.6	3.0
**Max Rotation [°]**	1.7	0.8	4.0	0.6	0.5	3.0

**Figure 1:**
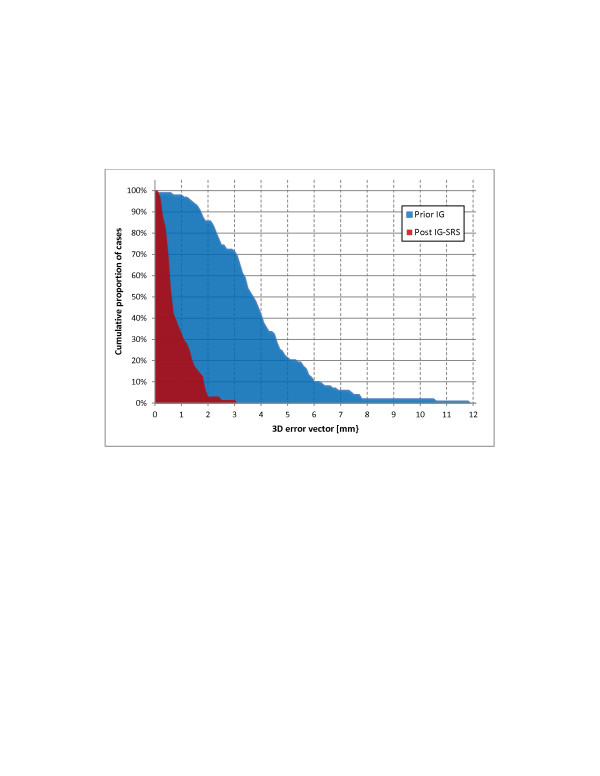
3D set-up errors observed after patient positioning and of 3D errors observed after image-guided radiosurgery: cumulative proportion of patients with 3D errors > xmm.

Errors observed after IG-RS were small and are summarized in Table 1. No systematic error ≥0.5 mm or ≥0.5° was observed in any direction and around any axis. The average intra-fractional 3D error was 0.9 mm ± 0.6 mm; intra-fractional errors were ≤1 mm and ≤2 mm in 67% and 97% of all cases, respectively. The distributions of 3D errors prior to IG and after IG-RS are shown in Figure 1. Rotations were ≤1° in 86% of the cases. Intra-fractional errors were not significantly different between the single-layer and double-layer masks with 1.0 mm ± 0.6 mm and 0.8 mm ± 0.6 mm (p = 0.34), respectively. There was no significant correlation between pre- and post-treatment 3D errors (p = 0.3). The median interval between pre- and post IG-RS cone-beam CT imaging was 23 min ranging between 15 min and 70 min. There was a significant correlation between this treatment time and intra-fractional errors: intra-fractional errors were 0.7mm±0.5mm and 1.2mm±0.7mm for treatment times ≤23 minutes and >23 minutes (p<0.01), respectively.

### Dosimetric accuracy of IG-RS

#### IG-RS without safety margins

**Figure 2:**
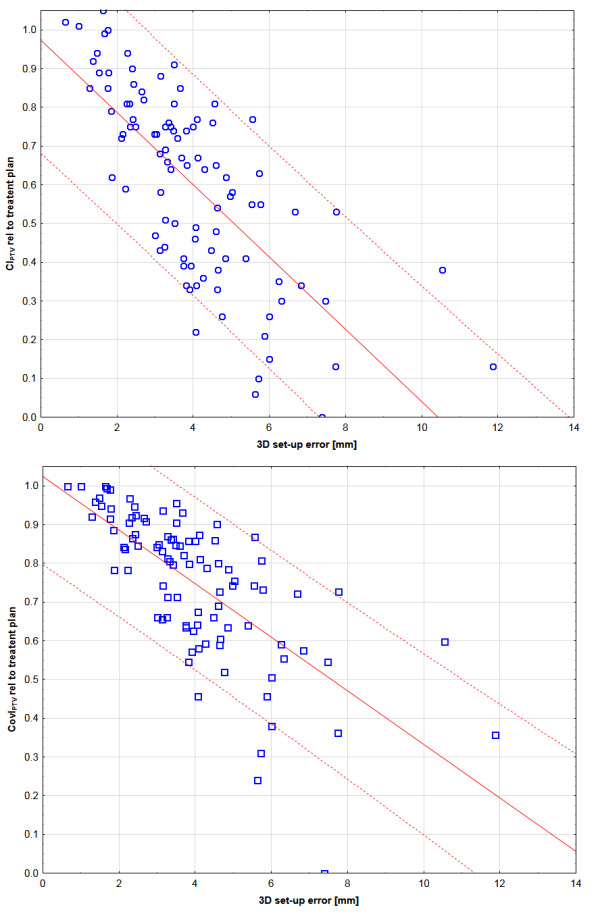
**Linear regression [solid line] and 90% prognosis [dashed line] between 3D set-up errors and changes of the dose distribution to the PTV: a) Paddick conformity index of the PTV (CI**_**PTV**_**): b) Coverage index of the PTV (CovI**_**PTV**_**).**

Simulation of treatment without IG decreased PTV coverage and conformity immediately and highly significantly; results are summarized in Tables 2 and 3. On average, combined translational and rotational set-up errors decreased CI_PTV_ to 60% ± 25% of the planned values and CovI_PTV_ to 75% ± 19%. Correlations between 3D set-up errors and changes of CI_PTV_ and CovI_PTV_ relative to the treatment plan were highly significant with r^2^ of 0.52 and 0.49, respectively (Figure 2). Each 3D set-up error of 1 mm decreased CI_PTV_ and CovI_PTV_ by 10% and 6% on average. The broad 90% prognosis range demonstrates large variability between the treatment plans: a 2 mm 3D set-up error reduced CI_PTV_ and CovI_PTV_ to 50%-100% and 66%-100% of the planned values, respectively.

**Table 2:** Paddick conformity index (CI) of the PTV (0 mm safety margins) 1) in the treatment plan (plan), 2) in the scenario of radiosurgery without image-guidance (No IG), 3) in the scenario of radiosurgery after image-guided correction of translational errors only and not rotations (IG trans) and 4) simulating errors observed immediately following image-guided radiosurgery (Post IG-RS)

	**Plan**		**No IG**		**IG trans**		**Post IG-SRS**	
**Absolute values**	0.73	±0.11	0.43	±0.18	0.73	±0.11	0.70	±0.11
**Values rel. to RT plan**	1	±0	0.60	±0.25	0.99	±0.03	0.97	±0.06

Absolute values and values relative to the corresponding treatment plans are summarized (average ± standard deviation).

**Table 3:** Dose distributions to the target with simulation of 0 mm, 1 mm and 2 mm safety margins 1) in the treatment plan (plan), 2) in the scenario of radiosurgery without image-guidance (No IG), 3) in the scenario of radiosurgery after image-guided correction of translational errors only and not rotations (IG trans) and 4) simulating errors observed immediately following image-guided radiosurgery (Post IG-RS)

**Safety margin**		**Plan**		**No IG**		**IG trans**		**Post IG-SRS**	
**0 mm**	**Absolute CI**	0.96	±0.06	0.72	±0.19	0.96	±0.06	0.94	±0.07
	**<95% planned CI**			91%		3%		14%	
**1 mm**	**Absolute CI**	1.00	±0.01	0.82	±0.19	1.00	±0.01	0.99	±0.01
	**<95% planned CI**			70%		0%		0%	
**2 mm**	**Absolute CI**	1.00	±0.04	0.90	±0.17	1.00	±0.04	1.00	±0
	**<95% planned CI**			40%		0%		0%	

Absolute values of the coverage index (average ± standard deviation) and percent of the patients with <95% planned target coverage are summarized.

Pre-treatment IG without correction of rotations but translations only did not decrease target coverage or conformity compared to the treatment plan (Tables 2 &3). A decrease of CI_PTV_ and CovI_PTV_ by >5% due to residual rotational errors was observed in 2 and 3 cases only, respectively.

Errors observed in CBCT imaging immediately following IG-RS decreased target coverage and conformity significantly, despite changes being small on average (Table 2 &3): the decrease of CI_PTV_ and CovI_PTV_ was <5% on average. A decrease of CI_PTV_ and CovI_PTV_ by >5% was observed in 23% and 14% of the cases, respectively; changes >10% were seen in <10% of the cases.

#### IG-RS with safety margins

**Figure 3:**
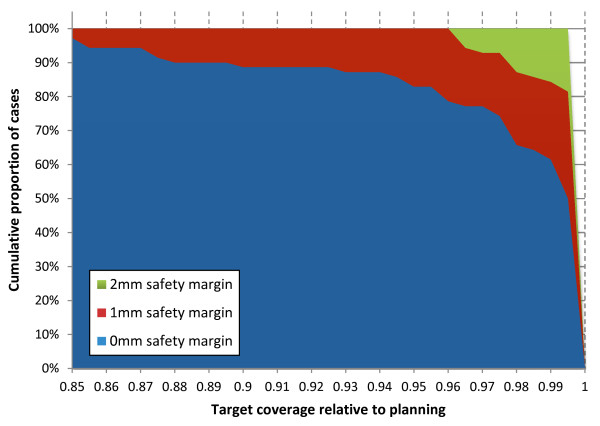
Target coverage relative to the treatment plan in the scenario of 0 mm, 1 mm and 2 mm GTV-to-PTV margins: cumulative proportion of patients with coverage >x%.

Safety margins decreased the detrimental effects of set-up errors but even a 2 mm GTV-to-PTV safety margin was not sufficient in the scenario of RS without IG: target coverage was 82% ± 19% and 90% ± 17% of the planned values on average after application of a 1 mm and 2 mm safety margin, respectively. In contrast, uncorrected pre-treatment rotational errors did not affect target coverage in a single patient after application of a 1 mm safety margin. Errors observed after IG-RS did not decrease target coverage by >5% in any patient, if a 1 mm safety margin was simulated. Results are summarized in Table 3 and Figure 3.

## Discussion

Several recent studies have analyzed patient set-up accuracy of different stereotactic mask systems and results vary considerably. Baumert et al. and Boda-Heggemann et al. reported 3D set-up errors between 3.1 mm and 3.7 mm on average for Scotch Cast masks and frame-based stereotactic positioning [10,11]. After frame-based stereotactic set-up in bite-block and / or thermoplastic masks, Masi et al. reported 3D errors of 2.1 mm to 2.9 mm on average [12]. Tryggestad et al. reported set-up errors between 2.1 mm and 2.7 mm for four different mask systems [13]. Wilbert et al. have recently described a novel semi-robotic positioning technique, which achieved promising accuracy of 1.6 mm [6]. The accuracy of the BrainLAB mask was 1.9 mm in the study by Gaevert et al. [14] and 0.5 mm when combined with a bite block as described by Minniti et al. [15]. In general, these set-up errors are larger compared to older studies [16-19]: this may be explained by the use of modern IGRT technologies, which allow more accurate detection of even small errors.

In our study, the 3D positioning errors was 3.9 mm ± 1.7 mm, which is at the upper end of the results in the literature. Two factors might explain this finding. Firstly, patient set-up was not based on stereotactic frames but based on alignment of the room lasers to conventional drawing marks on the masks; this concept is based on the experience that even frame-based stereotactic patient set-up requires online image-guidance to maximize accuracy [20]. Secondly, we observed a rather large systematic error of 2.7 mm in AP direction. The thermoplastic masks were made immediately prior to CT simulation and patients regularly reported a tighter fit of the thermoplastic mask at the time of treatment delivery. A complete hardening of the mask after CT simulation could push the patient deeper into the head mold and introduce this systematic error towards posterior.

All observed set-up errors were simulated within the treatment planning system and well established parameters for evaluation of radiosurgical dose distributions were analyzed [9,21]. Simulation of frame-less RS without image-guidance had detrimental effects on target coverage and dose conformity, which decreased by 25% to 40% on average, respectively. Steep dose gradients of RS treatment planning combined with 0 mm safety margins explain these large dosimetric effects of small set-up errors. These results clearly demonstrate the importance and necessity of image-guidance in radiosurgery: even safety margins of 1-2 mm were insufficient to compensate missing image-guidance and none of the frame-based stereotactic mask systems described above ensures an accuracy of patient set-up, where image-guidance would become redundant.

Despite consistent and uniform radiosurgical treatment planning, differences in target size, shape and location and consecutive differences in planned dose distributions resulted in large variability of the effect of set-up errors on target coverage and dose conformity. This underlines the importance of performing such simulation studies within the treatment planning system based on the patient individual dose distributions.

It is unclear whether image-guided correction of translational set-up errors is sufficient or whether additional correction of rotations further improves accuracy by a clinically relevant amount. In our study, target coverage and dose conformity were all within a 1% threshold on average compared to the treatment plan if image-guided correction of translations but not rotations was simulated. The limited relevance of rotational errors is explained by 1) the small target sizes, 2) location of the isocenter and image-guided correction reference point in the geometric center of the target and 3) spherical shape of the target volumes (metastases). The relevance of correcting rotational errors will certainly increase in larger and irregular shaped target volumes and especially when multiple lesions, which are located distant from each other, are treated with one isocenter.

A different finding was reported by Gevaert et al., where rotations decreased target coverage significantly and the authors recommended reducing lateral and longitudinal rotations to <0.5° [14]. Assuming a spherical target volume with a 2 cm diameter, a 0.5° rotation will move one point on the surface of the target volume by 0.09 mm; it is not understood, how such small errors can result in clinically significant changes of the planned dose distributions.

Intra-fractional patient motion was small in our study with <1 mm on average and no difference was observed between the single-layer and the more rigid double-layer mask. Based on these results, IG-RS is now routinely practiced with a single-layer thermoplastic mask at our department. These small errors measured after IG-RS represent a worst case scenario as intra-fractional motion is assumed to increase linearly with time [22]. This is confirmed by our results, where intra-fractional errors were significantly reduced from about 1.2mm to 0.7mm when the total treatment time was below the median of 23 minutes. Consequently, treatment times should be minimized as best as possible. The intra-fractional errors observed in our study are in very good agreement with data in the literature [6,8,11-14,23]. Dosimetric consequences of these intra-fractional errors were small on average but target coverage and dose conformity decreased by >5% in 14% and 23% of the patients, respectively, if 0 mm GTV-to-PTV safety margins were used. These results demonstrate that safety margins might be required despite highly accurate image-guided patient set-up: in our simulation study, a 1 mm GTV-to-PTV safety margin was sufficient so keep the dose to the GTV within a 5% threshold in all patients.

There are more uncertainties in the whole process of IG-RS than patient positioning and immobilization, e.g. image registration of planning CT and MRI, target definition and mechanical instabilities of the radiotherapy delivery machines. In “conventional” radiotherapy, we are used to compensating these uncertainties by application of sufficiently large safety margins e.g. using margin formulas [24]. Without sophisticated calculations available in literature, one can imagine that GTV-to-PTV margins of >2 mm are required in IG-RS when all these uncertainties are considered. However, this should be practiced with caution in single fraction IG-RS. The RTOG 90–05 study, which established radiosurgical doses, used conventional frame-based stereotactic techniques despite the treatment was most likely associated with all limitations as discussed above. Nevertheless, no safety margins were applied in the RTOG study and 0 mm PTV margins are considered as best practice of radiosurgery [25]. Prescribing the same doses in IG-RS as in the RTOG study will inevitably deliver higher doses to relevant volumes of normal tissue and potentially eloquent brain regions, if these doses are not prescribed to the GTV anymore but to a PTV after application of safety margins: the Paddick conformity index of the GTV was 0.73, 0.47 and 0.28 for 0 mm, 1 mm and 2 mm GTV-to-PTV safety margins. Such practice will increase the effective target dose to the GTV and potentially improve tumor control at the risk of increased toxicity. Clinical studies are need to evaluate the interplay effects between the accuracy of radiosurgical treatment, safety margins and irradiation dose and their influence on local control and toxicity.

When comparing the positioning accuracy and intra-fractional immobilization capacity of non-invasive IG-RS to traditional invasive stereotactic frames, it seems that intra-fractional patient motion is slightly larger with the non-invasive IG-RS approach [8]. On the other hand, recent studies suggest that the set-up using invasive frame-based stereotaxy is less accurate than previously assumed: image-guidance detected a systematic error of 1 mm in AP direction [23] and a frame slippage of 4.2 mm in 1/102 treatments of frame-based radiosurgery [8]. Consequently, it appears that the overall accuracy of non-invasive IG-RS is at least equivalent to invasive frame-based stereotaxy. Additional advantages of the non-invasive approach are absent risk of bleeding and infection and especially sufficient time for multi-modality imaging, multi-disciplinary target definition and complex treatment planning because of treatment planning and delivery are performed on different days [20].

## Conclusions

The accuracy of cranial radiosurgery is required to be within 1 mm or less to avoid decreased target coverage and dose conformality >5% compared to the treatment plan. Frame-less positioning of patients in thermoplastic masks results in substantial errors, which need to be corrected by online image-guidance. Image-guided correction of the translational error component seems to be sufficient with limited benefit of additionally correcting the rotational error component if the target volumes are relatively small, spherical shaped and the isocenter is located in the center of the target. The thermoplastic masks used in this study achieved effective immobilization such that coverage of the GTV with a 1 mm safety margin was not affected; keeping treatment times as short as possible will additionally minimize intra-fractional errors. Further clinical studies need to evaluate the interplay effects between the accuracy of radiosurgical treatment, safety margins and irradiation dose and their influence on local control and toxicity.

## Competing interests

The authors declare that they have no competing interest.

## Authors’ contributions

MG designed the study, collected the data and performed the data analysis. JR collected the data and performed the data analysis. KB and MF participated in the design of the study. RAS participated in the design of the study and data collection. All authors performed critical review of the manuscript and finally approved the manuscript.
